# Coupling and dynamics of cortical and autonomic signals are linked to central inhibition during the wake-sleep transition

**DOI:** 10.1038/s41598-017-09513-6

**Published:** 2017-09-18

**Authors:** Christine Ulke, Jue Huang, Justus T. C. Schwabedal, Galina Surova, Roland Mergl, Tilman Hensch

**Affiliations:** 1 Department of Psychiatry and Psychotherapy, University of Leipzig, Leipzig, Germany; 2 Max-Planck-Institute for the Physics of Complex Systems, Dresden, Germany; 3 Research Center of the German Depression Foundation, Leipzig, Germany

## Abstract

Maintaining temporal coordination across physiological systems is crucial at the wake-sleep transition. As shown in recent studies, the degree of coordination between brain and autonomic arousal influences attention, which highlights a previously unrecognised point of potential failure in the attention system. To investigate how cortical and autonomic dynamics are linked to the attentive process we analysed electroencephalogram, electrocardiogram and skin conductance data of 39 healthy adults recorded during a 2-h resting-state oddball experiment. We related cross-correlations to fluctuation periods of cortical and autonomic signals and correlated obtained measures to event-related potentials N1 and P2, reflecting excitatory and inhibitory processes. Increasing alignment of cortical and autonomic signals and longer periods of vigilance fluctuations corresponded to a larger and earlier P2; no such relations were found for N1. We compared two groups, with (I) and without measurable (II) delay in cortico-autonomic correlations. Individuals in Group II had more stable vigilance fluctuations, larger and earlier P2 and fell asleep more frequently than individuals in Group I. Our results support the hypothesis of a link between cortico-autonomic coupling and dynamics and central inhibition. Quantifying this link could help refine classification in psychiatric disorders with attention and sleep-related symptoms, particularly in ADHD, depression, and insomnia.

## Introduction

One of the main functions of the human brain is the co-regulation of cortical and autonomic arousal in adaptation to environmental and homeostatic requirements. In the process of falling asleep, cortical and autonomic arousal levels are gradually downregulated^1^, shielding the brain from irrelevant stimuli and promoting sleep onset^2,3^. Conversely, nocturnal awakenings, due to loud noise for example, are marked by sudden heart rate responses and changes in mental and attentional states. Thus, temporal coordination of different physiological modalities is crucial at state transitions^4^, but research on how theses subsystems communicate and to what extent their interplay influences attentional processes is still in an early stage.

While the dynamics of autonomic arousal levels can be tracked by observing the heart rate and skin conductance, levels of brain arousal as well as attentional processes can be observed in electroencephalographic recordings (EEG). The time course of brain arousal is reflected in spontaneous changes in the EEG, which we can automatically classify as 1-s EEG-vigilance stages^5–8^. Components of event-related potentials (ERP) visible in the EEG in response to sensory (e.g., auditory) stimuli reflect different functional processes. We discuss a negative component N1, centered around 100 ms after the stimulus onset^9^ and the following positive component P2^10^. Chait *et al*.^11^ as well as others^12–14^ observed enhanced P2 amplitude in response to actively ignored tones during a selective attention task. In such paradigms of selective attention or discrimination, P2 reflects inhibitory processes^11,13^ possibly modulating thresholds of conscious perception by suppressing interference from distracting stimuli^11,12^, thereby facilitating stimuli discrimination^15,16^ resulting in improved reaction times^15^. The component N1 is often considered a mostly exogenous component (related to stimulus properties), associated with the primary afferent excitation^9,17^; however the N1 amplitude was also shown to be modulated by (selective) attention to stimuli^18–21^.

Changes in brain arousal at the wake-sleep transition are reflected in altered responsivity of N1 and P2 to sensory stimuli^22–27^. Experiments in animals suggested that ERPs conform to the state of brain arousal via thalamo-cortical gating mechanisms modulating the neuronal activity of cortical areas^28–30^. Cortical activity depends on the balance between synaptic excitation and inhibition^31,32^. While the mechanism underlying the orchestration of different physiological modalities at state transitions is not entirely clear, it may depend on a waxing and waning of recurrent excitatory and inhibitory activity within cortical networks^31,33^.

An imbalance of these circuits has been implicated in psychiatric and neurological disorders^34–39^. Altered inhibitory and excitatory processes have been reported in disorders with sleep onset problems, such as depression and insomnia^36,40,41^, where symptoms of central hyperarousal^42–44^ and aberrant sleep-wake patterns^45,46^ are prevalent. EEG-derived measures of cortical activation and arousal regulation^47–49^ and cortical and autonomic co-regulation^47^ have been identified as possible biomarkers for prediction of antidepressant treatment response. A dysregulation of the locus coeruleus-norepinephrine (LC–NE) system—known to mediate cortical/ autonomic arousal and to prime neurons to stimuli response^50,51^—has been suggested^52–54^. In healthy individuals, a functional link between the LC–NE system and ERP component P3 (known to modulate with attention to a task) has been demonstrated^55,56^. Further, the degree of central-autonomic coupling has been associated with attentive behaviour^57^. These studies provide evidence, that the coupling of cortical and autonomic signals may impact attentional processes and the elucidation of their dynamics (i.e. signal fluctuations) may have clinical relevance.

In the present study we investigated how coupling of cortical and autonomic signals and their dynamics affect the attentive process during the wake-sleep transition. We hypothesise that: (H1) cortico-autonomic coupling, registered as correlation strength and delay, is linked to fluctuation periods of cortical and autonomic signals; (H2) cortico-autonomic coupling and dynamical fluctuations in the vigilance and autonomic state are linked to amplitude and latency of ERP components N1 and P2 as indices of auditory processing.

To test our hypotheses, we reanalysed a previously published data set^25^ in which 2-h EEG, electrocardiogram (ECG) derived heart rate (HR) and skin conductance (SCL) data were simultaneously recorded during the resting state, while tones were presented in an ignored oddball sequence. We computed time-lagged correlations of autonomic signals (HR, SCL) with a signal of cortical arousal, assessed by EEG-vigilance stages (V) using the Vigilance Algorithm Leipzig (VIGALL 2.1) and related their magnitude and delays to the fluctuation period of cortical and autonomic signals. Our analysis led us to distinguish individuals with (Group I) and without measurable delay (Group II). We further related this signature of cortico-autonomic coupling and fluctuations to stimuli-induced N1 and P2 amplitudes and latencies. Finally, we compared individuals with and without delay in cortico-autonomic correlations in regard to estimated fluctuation periods, ERP components N1 and P2, and the frequency of falling asleep during the 2-h resting EEG.

## Results

### Cortico-autonomic coupling: cross-correlations between cortical and autonomic signals

**Figure 1:**
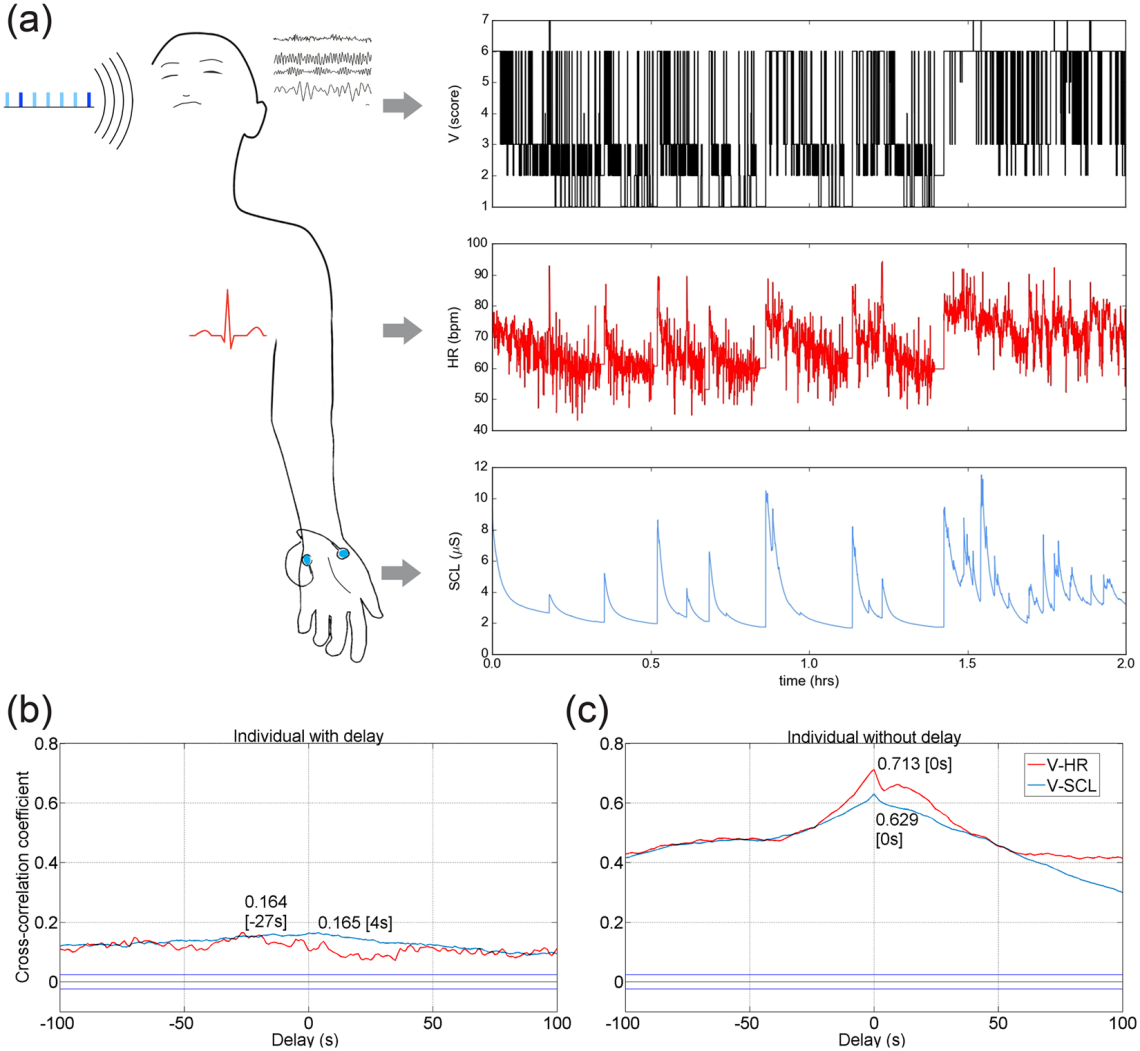
Time course of scored EEG-vigilance, heart rate and skin conductance level, and prototypical cross-correlations. (**a**) 2-h time series of scored EEG-vigilance (V, top row), heart rate (HR, middle row) and skin conductance level (SCL, bottom row) during an auditory oddball experiment in a single subject. (**b,c**) Cross-correlation coefficients between V-HR (red) and V-SCL (blue) over a range of ±100 s based on normalised values in an individual with **(b)** and without **(c)** delay between cortico-autonomic signals.

To assess the coupling between cortical signal V, and autonomic signals HR and SCL, we computed cross-correlations over a range of ±100 s. We determined the maximal cross-correlation coefficient for V and HR (C_VHR_), and V and SCL (C_VSCL_) time series in all subjects (N = 39; mean C_VHR_ = 0.362, range: −0.069 to 0.762; SD = 0.192; mean C_VSCL_ = 0.277; range: −0.299 to 0.629, SD = 0.211). We computed C_max_ as the mean of C_VHR_ and C_VSCL_ in absolute value. Further, we determined the delays at C_VHR_ (mean delay = −1.564 s, SD = 20.483) and C_VSCL_ (mean delay = −1.359 s, SD = 26.534). We computed Τ_max_ (Τ_max_ = 8.077 s, SD = 15.408, range: 0–49) as mean delay at C_VHR_ and C_VSCL_, in absolute value. A schematic overview of the recording, examples of V, HR and SCL time series and prototypical respective cross-correlations are presented in Fig. 1.

### Description of cortical and autonomic arousal fluctuations

**Figure 2:**
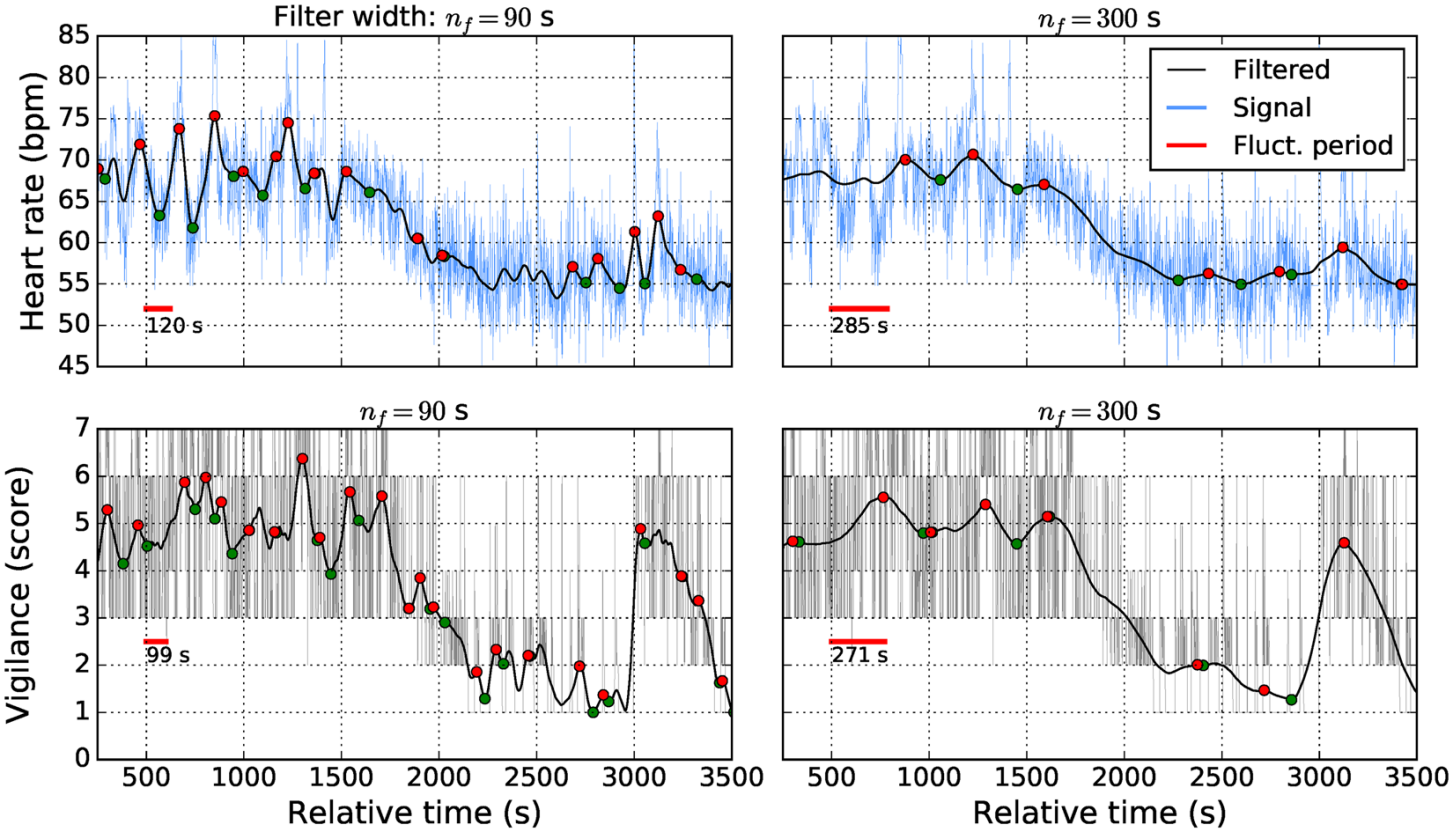
Fluctuation period analysis of heart rate (HR) and vigilance (V) signals at different filter widths. We show a typical segment of HR- and V-signals from one recording (thin lines), the filtered signals (thick lines) at indicated *n*_*f*_, and the local extrema (circles) abiding to the threshold condition. The median period (length of red bars) between consecutive local maxima (red circles), which we use as a statistical description of signal fluctuations, depends on the filter width *n*_*f*_ and the signal properties. Using narrow filters (left panels), fine-grained rhythms are described by the fluctuation period, while for increasing filter width slower rhythms are captured. HR (top panels) and V (bottom panels) signals differ in their smoothness visibly. Even though we apply formally the same algorithm to compute the period, the 90 s filter width leads to a very different period estimate, for example.

In the next step, we analysed how cortico-autonomic coupling is related to dynamical fluctuations in cortical and autonomic arousal states. The fluctuations were quantified by the median period between consecutive maxima in the signal. To avoid including spurious maxima, we only considered maxima in between which the overall amplitude exceeded a threshold (see methods/signal preprocessing and analysing). Furthermore, each signal was low-pass filtered with a normalised tent function, which focused the analysis on fluctuations within a specific time scale. For short tent widths (<10 s), the period of maxima reflected fast fluctuations governed by respiratory sinus arrhythmia in case of HR, for example. Broad filters (>200 s) lead to relatively few maxima within the 2 h recordings, that were governed predominantly by arousal reactions evoked by the experimenter (cf. Fig. 2). Within these bounds, a larger period indicated a greater temporal stability of vigilance and autonomic states.

### Cortico-autonomic coupling strength (C_max_) and fluctuations: correlation analyses

For each signal, the nominal value of fluctuation periods strongly depended on the used filter width, but for V and HR, the periods were consistent within a range of 10%. Conversely, the SCL-derived period at the same filter width was multiple times larger due to the intrinsically inert dynamic response of skin conductance. Because of this lack of response, we omitted SCL from the analysis of this section. We computed the median fluctuation period for a series of different filter widths between 10 and 200 s for the EEG-vigilance signal V and the autonomic signal HR. We correlated the fluctuation periods with the maximal correlation C_max_ between cortical and autonomic time series.

**Figure 3:**
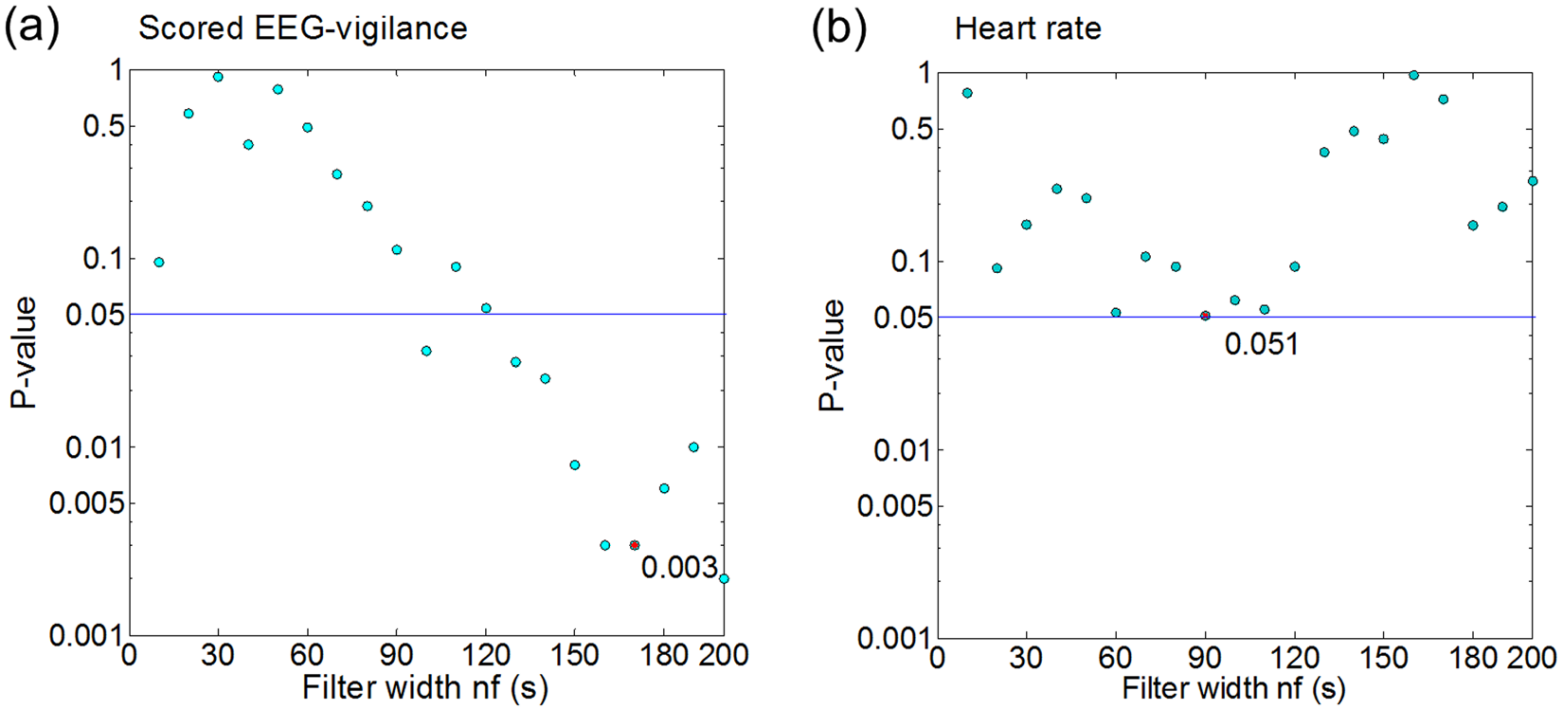
P-values for correlation between C_max_ and estimated fluctuation periods in function of width *n*_*f*_. (**a**) P-values for correlation between C_max_ and estimated fluctuation periods of scored EEG-vigilance (V). At a filter width of *n*_*f*_ = 200 s, the V-derived fluctuation period exceeded the 300 s bound in four individuals, possibly reflecting the wake-up reaction introduced by the experimenter (subjects who fell asleep were woken up after 300 s to keep the individual in a range of wakeful vigilance throughout the trial). Therefore, the filter width of *n*_*f*_ = 170 s was selected; (**b**) P-values for correlation between C_max_ and estimated fluctuation periods of heart rate.

V-derived periods (mean fluctuation period = 188.679 s, range: 118–261 s, SD = 35.579 s) were positively correlated with C_max_ showing a correlation of 0.469 (p = 0.003) at a filter width around 170 s (cf. Fig. 3(a)). HR-derived periods (mean fluctuation period = 107.423 s, range: 66–141.5 s, SD = 14.926 s) revealed a marginal negative correlation with C_max_ at a filter width around 90 s (r = −0.315, p = 0.051; cf. Fig. 3(b)). We used these filter widths in subsequent fluctuation analyses.

### Cortico-autonomic coupling delay (T_max_) and fluctuations: correlation analyses

Spearman correlation analyses revealed negative associations between V-derived fluctuation periods and T_max_ showing a correlation of rho = −0.466 (p = 0.003) at a filter width around 170 s. Concerning T_max_ and HR-derived periods, Spearman correlation analyses revealed a trend at a filter width around 90 s (rho = 0.294, p = 0.069). Due to the high occurrence of individuals with no measurable delay between time series (N = 20), we additionally performed group comparisons (cf. section *Group characteristics: individuals with and without cortico-autonomic coupling delay*).

### Cortico-autonomic coupling and sensory processing: correlation between Τ_max_ and ERPs

Next we investigated the association between cortico-autonomic coupling (Τ_max_) and the components N1 and P2 recorded during the oddball task. Due to the known frontocentral dominance of both, N1 and P2, mean amplitudes and latencies were analysed at electrode positions Fz and Cz. Spearman rank correlations coefficients were calculated due to the violation of normality of Τ_max_. Concerning the correlation between Τ_max_ and components N1 and P2, we found no significant correlations with either N1 latencies or N1 amplitudes to both deviant and standard stimuli (0.630 ≤ p ≤ 0.930). In contrast, Τ_max_ correlated negatively with P2 amplitudes to standard (Fz: rho = −0.495, p = 0.001, Cz: rho = −0.388, p = 0.015) and to deviant stimuli (Fz: rho = −0.605, p = 4.5*10^−5^, Cz: rho = −0.522, p = 6.5*10^−4^). In regard to the correlation between Τ_max_ and P2 latencies, we found a significant correlation for deviant stimuli at Cz (Fz: r = 0.280 p = 0.084, Cz: rho = 0.336, p = 0.037) but not for standard stimuli (Fz: p = 0.434; Cz: p = 0.717).

### Correlation between ERPs and V- and HR-derived fluctuation periods

**Figure 4:**
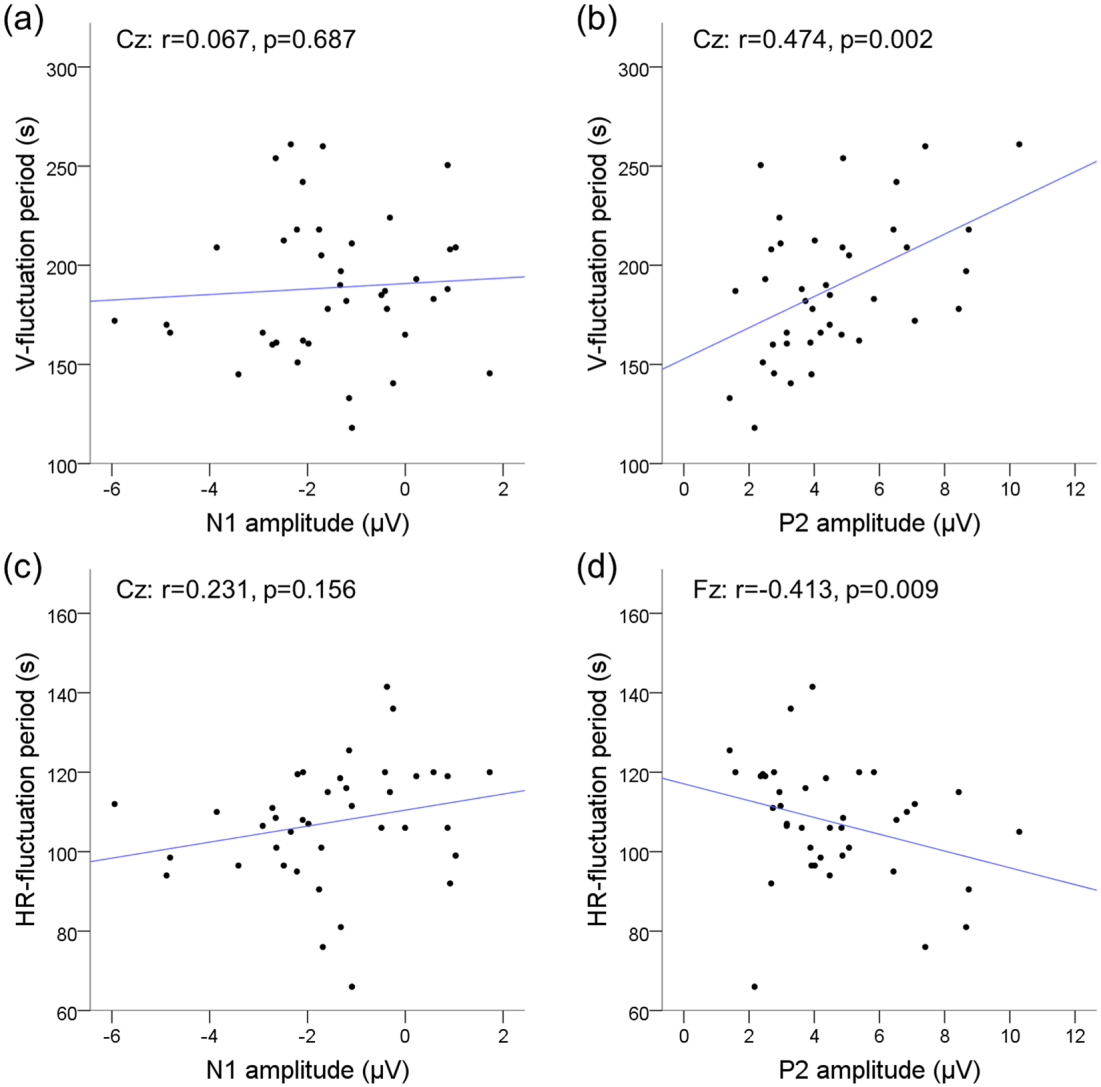
Correlations between event-related potentials (N1, P2) and median fluctuation period of EEG-vigilance signal V and heart rate HR. Correlations between (**a**) N1 amplitude to standard stimuli at Cz and V-derived fluctuation periods (filter width *n*_*f*_ = 170 s), (**b**) P2 amplitude to standard stimuli at Cz and V-derived fluctuation periods (*n*_*f*_ = 170 s), (**c**) N1 amplitude to standard stimuli at Cz and HR-derived fluctuation periods (*n*_*f*_ = 90 s), (**d**) P2 amplitude to standard stimuli at Fz and HR-derived fluctuation periods (*n*_*f*_ = 90 s).

Next, using Pearson’s product-moment correlation, we explored the relations between V- and HR-derived fluctuation periods and the ERPs (N1, P2). At Fz and Cz electrodes, we correlated the N1- and P2 responses with fluctuation periods computed. P2 amplitude to standard (Fz: r = 0.457, p = 0.003; Cz: r = 0.474, p = 0.002) and to deviant stimuli (Fz: r = 0.385, p = 0.015; Cz: r = 0.423, p = 0.007) correlated positively with V-derived fluctuation periods (cf. Fig. 4(b)). This finding indicates that enhanced P2 amplitudes are accompanied by temporal stability of V-fluctuation periods within a given bound. Concerning P2 latency, inverse correlations were found regarding deviant stimuli at Fz (r = −0.350, p = 0.029) and at Cz (r = −0.318, p = 0.049), whereas no correlation could be found for standard stimuli (0.627 ≤ p ≤ 0.740).

By contrast, P2 amplitude to standard (Fz: r = −0.413, p = 0.009; Cz: r = −0.303, p = 0.060) and deviant stimuli (Fz: r = −0.421, p = 0.008; Cz: r = −0.306, p = 0.058) was negatively correlated with HR-derived fluctuation periods (cf. Fig. 4(d)). This may indicate that, within a certain bound, a stronger stability of HR-derived fluctuation periods relates to smaller P2 amplitudes. Concerning P2 latency, no correlation could be found (0.211 ≤ p ≤ 0.939). In regard to N1, no significant correlations were found with V-derived (0.302 ≤ p ≤ 0.998) or with HR-derived (0.115 ≤ p ≤ 0.513) fluctuation periods (cf. Fig. 4(a and c)).

### Correlation between P2 amplitude and the frequency of falling asleep

To assess the association of P2 amplitude with sleep onset behavior, under control of the arousal level, we correlated P2 amplitude─restricted to EEG-vigilance stage A1─with the frequency of falling asleep (mean = 2.97; range: 0 to 8, SD = 2.0). EEG-vigilance stage A1 was selected, since it occurred most frequently among all subjects during the 2-h recording. P2 amplitudes to standard (Fz: rho = 0.563, p = 1.9*10^−4^; Cz: rho = 0.491, p = 0.002) and deviant stimuli (Fz: rho = 0.478, p = 0.002; Cz: rho = 0.362, p = 0.043) were positively correlated with the frequency of falling asleep. No such relations were found for N1 amplitude (0.209 ≤ p ≤ 0.360).

### Group characteristics: individuals with and without cortico-autonomic coupling delay

Dependent on Τ_max_, subjects were partitioned into two groups: Group I (with delay, Τ_max_ ≥ 1 s); Group II (without measurable delay, Τ_max_ < 1 s). Group characteristics are presented in Table 1. Group differences did not reach significance level (p < 0.05) concerning demographics, EEG-relevant measures, and autonomic measures (Table 1). Groups differed significantly regarding the mean vigilance score (t_37_ = 2.613, p = 0.017, Cohen’s *d* = 0.86), the percentage of switches between main vigilance stages (t_30_ = 2.541, p = 0.017, Cohen’s *d* = 0.85), and the frequency of falling asleep (t_37_ = −2.487, p = 0.018, Cohen’s *d* = 0.82). In addition, V-derived fluctuation periods at *n*_*f*_ = 170 s were observed to be different between Groups I and II (t_37_ = −3.255, p = 0.002, Cohen’s *d* = 1.07), wherein individuals without delay (Group II) showed longer periods than Group I. HR-derived periods were not different between groups.

**Table 1:** Characteristics of Group I (with delay, N = 19) and Group II (without delay, N = 20).

Measures		Group with delays (N = 19)	Group without delays (N = 20)	p
Demographics				
Sex	f (%)	9 (47.4)	13 (65)	0.267
	m (%)	10 (52.6)	7 (35)	
Age (yrs)	mean (SD)	25.1 (4.1)	22.8 (3.5)	0.073
	range	18–33	18–31	
EEG-relevant measures				
Coffee consume (ml/day of recording)	mean (SD)	121.1 (139.8)	70.0 (137.1)	0.257
Tee consume (ml/day of recording)	mean (SD)	44.7 (94.1)	40.0 (127.3)	0.896
KSS before recording	mean (SD)	5.8 (1.8)	6.2 (1.5)	0.446
	range	3.0–8.0	3.0–8.0	
KSS after recording	mean (SD)	4.5 (2.2)	5.1 (2.2)	0.384
	range	1.0–8.0	1.0–8.0	
Cortical arousal (2-h EEG)				
Mean vigilance score	mean (SD)	5.0 (0.6)	4.4 (0.8)	0.017
	range	3.8–6.1	2.6–5.4	
Switches between main stages^a^	% (SD)	17.4 (9.5)	10.9 (5.9)	0.017
ANS measures				
HR (beats/min)	mean (SD)	62.4 (6.9)	63.6 (6.9)	0.623
	range	50.2–76.2	53.7–80.7	
SCL (µSiemens)	mean (SD)	3.5 (4.4)	2.7 (2.8)	0.518
	range	0.6–19.6	0.5–12.6	
Frequency of falling asleep (2-h EEG)				
	mean (SD)	2.2 (2.0)	3.7 (1.7)	0.018
	range	0–7	1–8	
Cortico-autonomic coupling				
C_VHR_	mean (SD)	0.3 (0.2)	0.5 (0.1)	1.4*10^−4^
	range	−0.1–0.5	0.2–0.8	
C_VSCL_	mean (SD)	0.1 (0.2)	0.4 (0.1)	1.4*10^−5^
	range	−0.3–0.4	0.2–0.6	
Fluctuation period				
Estimated mean periods (s) for V	mean (SD)	171.7 (25.3)	204.8 (36.9)	0.002
	range	133.0–224.0	118.0–261.0	
Estimated mean periods (s) for HR	mean (SD)	111.3 (10.7)	103.7 (17.5)	0.111
	range	96.5–136.0	66.0–141.5	

Groups were partitioned dependent on Τ_max_: Group I (Τ_max_ ≥ 1 s); Group II (Τ_max_ < 1 s).

SD = standard deviation; KSS = Karolinska Sleepiness Scale; ANS = autonomic nervous system; HR = heart rate; SCL = skin conductance level; V = EEG-vigilance; ^a^empirically determined percentage of stage switches between stage 0, A (A1, A2, A3), B (B1, B2/3) and C.

### Group comparison of ERP parameters N1 and P2

**Figure 5:**
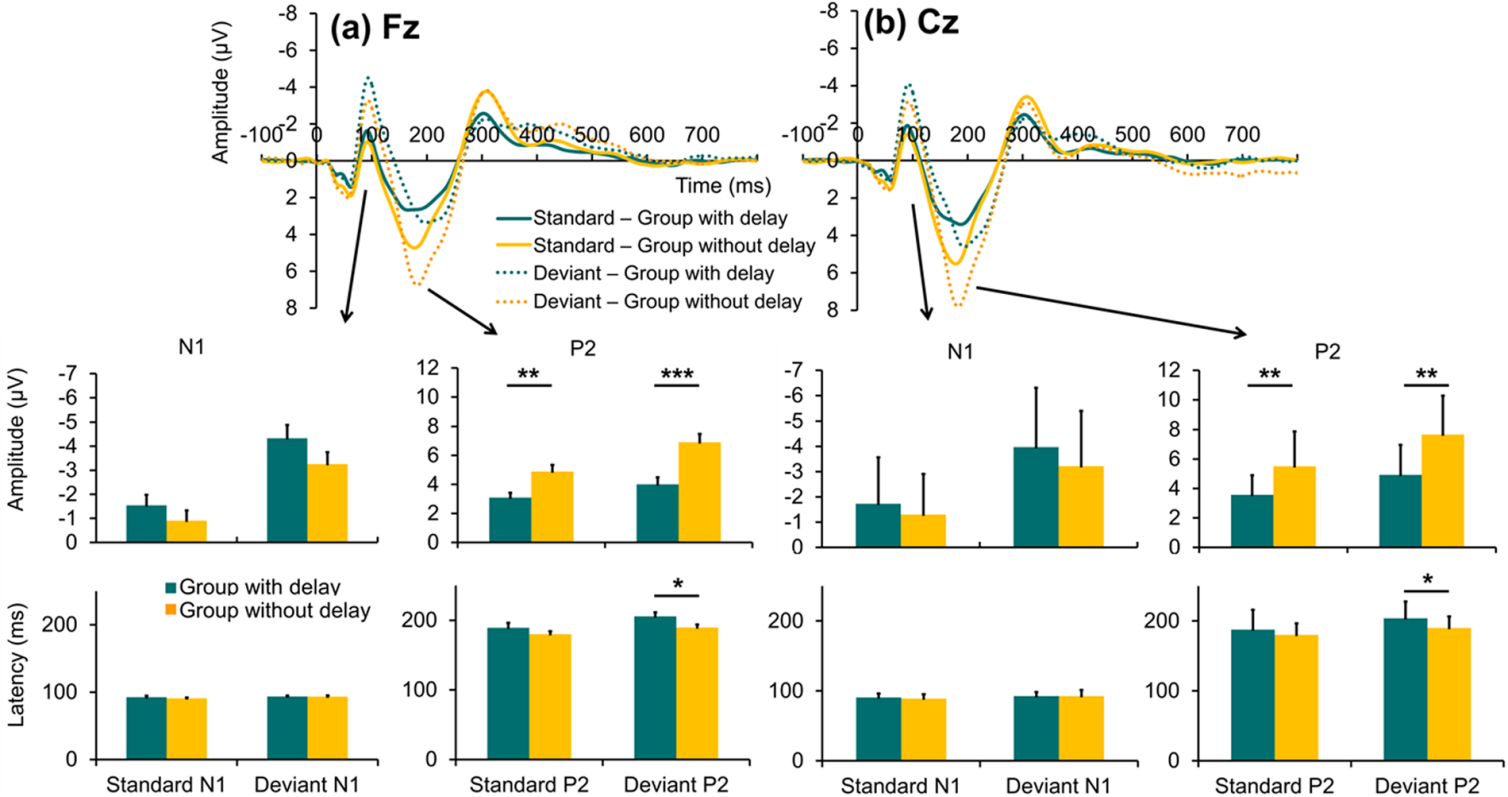
Comparisons of ERP grand average between groups. N1 and P2 to standard and deviant stimuli at Fz (left) and Cz (right) are illustrated in grand average waveforms. Amplitudes and latencies of each component based on individual peaks are presented in bar charts. Error bars reflect standard error of the mean. Significant results of group comparisons are marked with asterisks.

There was no significant group difference in N1 amplitudes (0.153 ≤ p ≤ 0.445) or latencies (0.435 ≤ p ≤ 0.990) to both standard and deviant stimuli (cf. Fig. 5). In contrast, subjects in Group I displayed a smaller P2 amplitude to both standard (Fz: t_37_ = −3.615, p = 0.003, Cohen’s *d:*1.022; Cz: t_30.235_ = −3.191, p = 0.003, Cohen’s *d*: 1.013) and deviant stimuli (Fz: t_37_ = −3.875, p = 4.2*10^−4^, Cohen’s *d:*1.245; C: t_37_ = −3.628, *p* = 0.001, Cohen’s *d*: 1.165) compared to subjects in Group II (cf. Fig. 5). Concerning P2 latencies, we found significant group differences for deviant stimuli (Fz: t_37_ = 2.224, p = 0.032, Cohen’s *d*: 0.710; Cz: t_37_ = 2.132, p = 0.040, Cohen’s *d*: 0.679), wherein subjects in Group I had longer latencies than subjects in Group II. No significant group differences were found for mean P2 latencies to standard stimuli (Fz: p = 0.289; Cz: p = 0.327).

### Control analyses for group difference of P2

To control the group differences in P2 amplitudes and latencies for arousal levels, we calculated and compared P2 amplitudes and latencies in Group I and II restricted to EEG-vigilance stage A1. EEG-vigilance stage A1 was selected, since it occurred most frequently among all subjects during the 2-h recording. Clear differences between Group I and II concerning P2 amplitudes and (partly) latencies could be demonstrated. Detailed results are presented in Table 2.

**Table 2:** Mean amplitudes and peak latencies of N1 and P2; Group I (with delay: N = 19) and Group II (without delay: N = 20) in EEG-vigilance stages A1. Groups were partitioned dependent on Τ_max_: Group I (Τ_max_ ≥ 1 s); Group II (Τ_max_ < 1 s).

Location		Group with delays (N = 19)	Group without delays (N = 20)	t (df)	p	Cohen’s *d*
	amplitude (µV)					
Fz	standard N1	−2.27 (1.99)	−2.23 (2.27)	t_(37)_ = −0.056	0.955	0.019
	deviant N1	−5.05 (2.51)	−5.05 (2.55)	t_(37)_ = 0.148	0.883	0.000
	standard P2	3.26 (1.33)	5.18 (2.71)	t_(27.944)_ = −2.843	0.008	0.899
	deviant P2	4.33 (2.22)	6.99 (2.72)	t_(23.607)_ = −2.795	0.010	1.071
Cz	standard N1	−2.41 (1.97)	−2.51 (2.18)	t_(37)_ = 1.439	0.159	0.048
	deviant N1	−4.54 (2.64)	−4.69 (2.65)	t_(37)_ = 0.177	0.861	0.057
	standard P2	3.82 (1.13)	6.00 (3.29)	t_(37)_ = 2.981	0.005	0.886
	deviant P2	5.30 (2.11)	8.15 (3.20)	t_(33.053)_ = −3.290	0.002	1.052
	latency (ms)					
Fz	standard N1	92.68 (7.48)	89.55 (6.09)	t_(37)_ = 1.439	0.159	0.176
	deviant N1	94.05 (8.05)	92.90 (8.69)	t_(37)_ = 1.182	0.245	0.137
	standard P2	186.79 (31.87)	189.50 (20.91)	t_(30.848)_ = −0.312	0.757	0.101
Cz	deviant P2	213.58 (27.72)	190.85 (19.37)	t_(37)_ = 2.981	0.005	0.951
	standard N1	91.74 (7.53)	89.10 (6.38)	t_(37)_ = 0.429	0.670	0.378
	deviant N1	92.68 (7.02)	92.05 (8.17)	t_(37)_ = 0.260	0.797	0.083
	standard P2	186.79 (27.04)	187.60 (21.89)	t_(37)_ = −0.103	0.918	0.033
	deviant P2	207.74 (29.89)	191.95 (17.14)	t_(28.378)_ = 2.010	0.054	0.648

μV = microvolt; ms = milliseconds. Standard deviations are presented in parentheses.

## Discussion

Functional states are coordinated across the networked physiological systems of the body to facilitate reliable and appropriate responses of organism-wide functions, as well as transitions between physiological states when falling asleep, for example. Possibly, some dysfunctions are well-detectable through quantitative changes in the links coordinating different physiological sub-systems. To explore this line of inquiry, we investigated the correlations between cortico-autonomic coupling and dynamical fluctuations, and their relation to indices of auditory processing at the wake-sleep transition. In line with Hypothesis 1 (H1), we found correlations between C_max_ (the maximal cross-correlation of cortical and autonomic signals), T_max_ (the individual delay) and V-derived fluctuation periods (p = 0.003) whereas the correlation between either C_max_ or T_max_ and HR-derived periods just failed to reach significance (C_max_: p = 0.051, T_max_: p = 0.069). In line with Hypothesis 2 (H2) we found that increasing temporal alignment of cortical and autonomic signals corresponded to a larger and earlier P2. When correlating P2 with average periods of V- and HR-derived fluctuations, we observed that longer periods of V-derived fluctuations corresponded to an earlier and larger P2, whereas HR-derived fluctuation periods were negatively correlated with P2 amplitudes. This suggests that both cortico-autonomic coupling and dynamical fluctuations in the vigilance and autonomic state are linked to cortical inhibitory processes. Interestingly, no such relationships could be established in regard to N1, suggesting that cortical and autonomic coupling and their associated dynamics are not linked to cortical excitatory processes (H2).

States of sleep and mental alertness can be disrupted by sudden changes in arousal. Conversely, a tight coordination of autonomic and cortical processes should stabilise such physiological states. It is therefore not surprising that dynamical coupling has been revealed among autonomic and cortical signals with varying magnitude and time delay^1,58–61^. In the present study, we specifically investigated to what degree such cortico-autonomic coupling, registered as correlation strength and delay, affects the stability of wake-sleep transitions. We found that a larger coupling strength and shorter delay predicts significantly longer average periods of vigilance fluctuations on a time-scale of about 190 s. In addition, individuals in Group II with shorter coupling delays exhibited significantly longer periods of vigilance fluctuations in comparison to individuals in Group I meaning that vigilance states were more stable in individuals with shorter coupling delays.

To better understand the physiological mechanism underlying the relationship of coupling and vigilance stability, we correlated cortico-autonomic delays with ERP responses. Short delays corresponded with an earlier and more pronounced ERP component P2, but not N1. We considered the possibility that this relationship resulted from shifts in the wake-sleep state which are known to alter ERP responses^22,24,25,62^. However, even when restricting the analysis to EEG-vigilance stage A1, associated with relaxed wakefulness and dominant alpha activity at posterior sites, individuals without measurable cortico-autonomic delay had a larger and substantially earlier P2 than individuals in Group I. In addition, a dominant state-shift effect on ERP responses would have likely impacted not only P2 but also N1, known to linearly decrease with descending levels of vigilance^22,25^. Under the assumption that P2 amplitude indicates the strength of cortical inhibitory processes when stimuli are ignored^11–13^, this finding further substantiates our hypothesis that tighter cortico-autonomic coupling correlates with stronger cortical inhibition. Our results also suggest that cortico-autonomic coupling at the wake-sleep transition affects N1 and P2 independently, supporting the view of P2 as functionally distinct entity^10^. This is consistent with earlier studies showing that N1 coincides with an early^9^ and P2 with a later attentive^10,63^ stage of auditory processing.

Taken together, our findings hint at the involvement of cortico-thalamic networks in binding cortical arousal control. We observed V-derived fluctuation periods in the infra-slow range, about 0.005 Hz across all participants. Fluctuations in this range have been demonstrated *in vitro* in nuclei of the dorsal thalamus, posited to be driven by non-neuronal astrocyte activity^64^. While thalamo-cortical projections are predominantly excitatory, the thalamo-cortical loop is completed by inhibitory cortico-thalamic projections^31,65^. Increased period at stronger inhibition, as we found in our data, is in line with theoretical investigations that systems coupled through strong suppressive synaptic coupling show an increased stability in their dynamics^66^. Cortico-autonomic coupling could therefore be an interesting parameter to investigate in psychiatric conditions in which a derailed central inhibitory system has been implied, such as depression and ADHD^37–39^ or certain sleep disorders^67,68^.

In contrast to cortical vigilance, we obtained a negative correlation between HR-derived fluctuation periods and P2 (cf. Fig. 4) wherein shorter HR-derived fluctuation periods correlated with larger P2 amplitudes. When considering the frequency range, we found fluctuation periods between 66 s and 141.5 s across all individuals, relating to frequencies between 0.007 and 0.015 Hz. Signaling in this frequency range has been associated with cardiac function and blood flow^69^, specifically intrinsic myogenic activity of the vessel wall, controlling vasodilatation. Mechanisms of endothelial function including nitric oxide and endothelium-derived hyperpolarising factor are hypothesised to be involved in the regulation of this frequency range^70–72^. Interestingly, a recent study of simultaneous EEG/fMRI measurements found increased BOLD signal in temporal cortices but decreased BOLD signal in thalamic areas in lower stages of vigilance compared to vigilance stage A1^73^. Our findings possibly relate to the hemo-neural hypothesis^74^ and may point to a link between endothelial mechanisms of vasodilatation and inhibitory processes which remain to be further investigated in future studies.

It is conceivable that the degree of cortico-autonomic coordination affects inhibitory functions necessary for overt behavior. Breeden *et al*.^57^ examined whether the coupling of spontaneous fluctuations in brain and autonomic activity predicts individual differences in attentive behavior. They reported a positive coupling between pupil diameter and brain activity in regions of the cingulo-opercular network; individuals with tighter central-autonomic coupling were less prone to distractibility. Interestingly, decreased inhibitory control in insomnia patients in comparison to healthy controls were reported in a recent study by Cortoos *et al*.^41^. The authors found no differences in N1 amplitudes between insomnia patients experiencing sleep disruptions and good sleepers but instead disinhibition of information processing as evidenced with decreased P2 amplitudes in the insomnia group. In the present study, individuals in Group II, with no measurable delay between cortico-autonomic signals, fell asleep more frequently than those in Group I. In addition, larger P2 amplitude corresponded to higher frequency of falling asleep; no such relations were found for N1. These results suggest that inhibitory, more so than excitatory processes have an impact on sleep onset behavior which has been pointed out by Saper *et al*.^75^.

In conclusion, our study discloses an interesting link among cortico-autonomic coupling, EEG-vigilance and HR dynamics and central inhibition, within the diversity of healthy individuals studied here. Given the previously implied inhibitory impairments in psychiatric disorders of ADHD, depression or sleep disorders, our results indicate a possible clinical relevance of cortico-autonomic coupling which should be further investigated as potential biomarker in clinical studies.

## Methods

### Subjects

Healthy volunteers were recruited via local and online advertisements. Subjects without a history of psychiatric or neurological disorder or current intake of psychotropic medication were included. Exclusion criteria have been described in detail elsewhere^25^. The final sample included 39 subjects (22 females, age = 23.90 ± 3.93 years, age range 18–33 years). The study was approved by the local ethics committee of the University of Leipzig (075-13-11032013). Each subject gave written informed consent prior to the recording. All experiments were performed in accordance with relevant guidelines and regulations. All subjects received 20€ or course credits (psychology students) for their participation.

### EEG, heart rate and skin conductance data acquisition

The 2-h EEG recording sessions were held between 1 and 4 pm. During the EEG-preparation, participants were asked to complete questionnaires including a substance consumption scale, and the Karolinska Sleepiness Scale^76^. EEGs were recorded in a dimmed and sound-attenuated booth with a temperature below 25 degrees Celsius. The EEG was recorded with Ag/AgCl electrodes using a QuickAmp amplifier (Brain Products GmbH, Gilching, Germany) from 31 electrode positions (Fp1, Fp2, F3, F4, F7, F8, Fz, FC1, FC2, FC5, FC6, C3, C4, T7, T8, Cz, FT9, FT10, CP5, CP6, TP9, TP10, P3, P4, P7, P8, Pz, O1, O2, PO9, PO10) according to the extended international 10–20 system, referenced against the common average. Impedance of each electrode was kept below 10kΩ. A bipolar electrode placed lateral of the left and right eye served to monitor horizontal eye movements. Another bipolar electrode was placed above and below the right eye to monitor vertical eye movements. To acquire R-R intervals, an ECG was measured from two adhesive electrodes placed on both arms and recorded as a bipolar channel of a QuickAmp amplifier. To acquire the skin conductance level (SCL), a 0.5V-potential was applied to the non-dominant hand, and the current was recorded from another bipolar channel of the QuickAmp amplifier (GSR module, Brain Products GmbH, Gilching, Germany). The two electrodes (13mm diameter) were placed at the thenar and hypothenar. All data were sampled at a rate of 1000 Hz.

### Experimental paradigm

At the beginning of the experiment, the body position was changed from an upright to a laid-back position by adjusting the reclining chair. During the 120-min EEG, ECG, and SCL recording, subjects lay comfortably on the lounger with closed eyes while two tones (500 and 1000 Hz) were presented in an oddball sequence (interstimulus interval: 900–1400 ms) with stimuli probability of 80% and 20% respectively^77^, stimuli duration of 50 ms, with a rise and fall time of 10 ms and an intensity of 70 dB SPL. Stimuli were presented binaurally via insert earphones (E-A-RTONE 3 A, Aearo Company Auditory System, Indianapolis, IN, USA) using presentation software (Presentation, Neurobehavioral Systems). Subjects were instructed to ignore the stimuli, to relax and explicitly allowed to follow their own natural course of wakefulness decline. In case of the appearance of sleep-typical graphoelements (sleep spindles, K-complexes) during the EEG recording, the subjects were woken up after five minutes and asked to answer a simple question. This process was repeated until the end of the experiment.

### EEG-vigilance staging and processing

The 2-h EEG was analysed using BrainVision Analyzer software (Brain Products GmbH, Gilching, Germany). First, the raw EEG was pre-processed according to Standard Operating Procedure (SOP; see VIGALL manual, http://research.uni-leipzig.de/vigall/). After that, all 1-s artefact-free EEG-segments were classified into seven different EEG-vigilance stages using VIGALL 2.1 (see VIGALL manual, available at http://research.uni-leipzig.de/vigall/). The EEG-vigilance stage-scoring is presented in Table 3. Detailed staging and processing of this data has previously been published^25^.

**Table 3:** Assessment of EEG-vigilance stages by applying VIGALL 2.1.

VIGALL stages	Stage Scoring	Corresponding behavioural state	EEG-characteristics
0	7	cognitively active wakefulness	low amplitude, desynchronised non-alpha EEG without horizontal SEM
A1	6	relaxed wakefulness	occipital dominant alpha activity
A2	5		starting shifts of alpha to central and frontal cortical areas
A3	4		continued frontalisation of alpha
B1	3	drowsiness	low amplitude, desynchronised EEG with horizontal SEM
B2/3	2		dominant delta- and theta-power
C	1	sleep-onset	occurrence of K-complex and sleep spindles

VIGALL = Vigilance Algorithm Leipzig; EEG = electroencephalogram; SEM = slow eye movements.

### HR and SCL processing

Segments marked as artefacts in the EEG were marked automatically as artefacts in ECG and SCL channels. Only segments without artefacts went into further analysis. In the ECG recording, the ‘R’ peaks of the QRS complex were marked using an integrated algorithm in the BrainVision Analyzer software. The detection results were visually checked and corrected if necessary. HR was calculated from the mean of R-R intervals (60,000/R-R interval in ms) across three consecutive artefact free segments. SCL was computed as mean of all data points (100 sampling rate) in each EEG segment.

### Signal preprocessing and analysing

In a first step, we replaced missing values in the 2-h time series with a 1-sec resolution for EEG-vigilance (V), HR and SCL measures. Missing values were replaced due to artefacts by respective means (i.e., mean V, mean HR and mean SCL) of each subject for the corresponding time series. Thus, for each subject, three continuous time series were obtained. EEG-vigilance (V) and autonomic (HR, SCL) time series were normalised and cross-correlated over a range of ±100 sample delays (s) by using the function *crosscorr* in Matlab (MATLAB 6.1, The MathWorks Inc., Natick, MA, 2000). We quantified fluctuations in each signal by computing an effective period after low-pass filtering. To filter, we first convolved a signal with a normalised tent function of length *nf*. After this we determined cycle-wise periods and amplitudes but determining all maxima and minima. Amplitude was computed by averaging differences between a maximum and the last and following minimum. Only those cycles were considered further, which showed amplitudes larger than the median of all amplitudes. At last, we computed the average fluctuation period for a signal at filter width *nf* as the median of all considered periods. All software for this analysis was written in Python by one of the authors, J.T.C.S. (Fluctuation period of a signal with a certain filter width · GitHub).

### Auditory event-related potentials N1 and P2

The EEG data were analysed offline and a bandpass filter of 0.5–30 Hz was applied. Epochs of 900ms (100 ms pre- and 800 ms post-stimulus), time locked to the onset of each auditory stimulus, were averaged for each subject. Standard stimuli that immediately followed a deviant stimulus were excluded from analysis. Epochs with amplitudes exceeding ±100 µV were rejected. Baseline correction was applied for the 100ms pre-stimulus interval. A minimum of 50 epochs for each subject was necessary for averaging. Considering all EEG-vigilance stages, we obtained 3353.4 ± 312.95 and 1103.23 ± 111.78 (mean ± SD) epochs in response to standard and deviant stimuli, respectively. Considering EEG-vigilance stage A1, we obtained 872.54 ± 438.59 and 284.78 ± 146.40 (mean ± SD) epochs in response to standard and deviant stimuli, respectively. Subsequently grand averages respective for standard and deviant stimuli were executed at Fz and Cz. Individual peaks were detected by Vision Analyzer’s inbuilt peak detection module (semiautomatic detection, searched for local maxima) based on search windows derived from visual inspection of grand average waveforms. Then, the mean amplitude values within given windows around the identified peaks and the identified peak latencies by each individual were exported for each component (N1: +/−10ms; P2: +/−20ms) for statistical analyses.

### Statistical analysis

Before statistical analysis, we checked for homogeneity of variances and normality. To test H1 and H2, we calculated Pearson and Spearman correlation coefficients (the latter ones in case of non-normal distributions across all subjects). To assess group differences concerning age, gender, EEG-relevant measures, mean vigilance, EEG-vigilance stage switches, mean HR, mean SCL, frequency of falling asleep, fluctuation periods and amplitudes and latencies of components N1 and P2, we conducted independent t-tests, Qui-square tests (gender) and Mann-Whitney U tests (in case of non-normally distributed or ordinal data). Testing was carried out at the 0.05 significance level. Power analyses were conducted in G*Power. Given *N* = 39 and α = 0.05, power calculations revealed a required true effect size of *r* = 0.432 for correlation analyses or *d* = 0.922 for t-tests, respectively, to be detected with a chance of 80% (1-β). All statistical analyses were conducted in Matlab (MATLAB 6.1, The MathWorks Inc., Natick, MA, 2000) and SPSS (IBM SPSS Statistics version 24 (IBM, Armonk, NY, USA))

### Data availability statement

The authors declare that they comply with Nature’s policies of data availability.

## References

[1] Olbrich S (2011). Brain and body. Journal of Psychophysiology.

[2] Merica H, Fortune RD (2004). State transitions between wake and sleep, and within the ultradian cycle, with focus on the link to neuronal activity. Sleep medicine reviews.

[3] Ogilvie RD (2001). The process of falling asleep. Sleep medicine reviews.

[4] Bashan A, Bartsch RP, Kantelhardt JW, Havlin S, Ivanov PC (2012). Network physiology reveals relations between network topology and physiological function. Nature communications.

[5] Loomis AL, Harvey EN, Hobart G (1937). Cerebral states during sleep, as studied by human brain potentials. Journal of experimental psychology.

[6] Roth B (1961). The clinical and theoretical importance of EEG rhythms corresponding to states of lowered vigilance. Electroencephalography and Clinical Neurophysiology.

[7] Bente, D. Vigilanz, dissoziative Vigilanzverschiebung und Insuffizienz des Vigilitätstonus. *Begleitwirkung und Mißerfolge der psychiatrischen Pharmakotherapie*, 13–28 (1964).

[8] Hegerl, U. *et al*. Vigilance Algorithm Leipzig (VIGALL) Version 2.1 – Manual, http://research.uni-leipzig.de/vigall/ (2016).

[9] Näätänen R, Picton T (1987). The N1 wave of the human electric and magnetic response to sound: a review and an analysis of the component structure. Psychophysiology.

[10] Crowley KE, Colrain IM (2004). A review of the evidence for P2 being an independent component process: age, sleep and modality. Clinical neurophysiology.

[11] Chait M, de Cheveigné A, Poeppel D, Simon JZ (2010). Neural dynamics of attending and ignoring in human auditory cortex. Neuropsychologia.

[12] Melara RD, Rao A, Tong Y (2002). The duality of selection: excitatory and inhibitory processes in auditory selective attention. Journal of Experimental Psychology: Human Perception and Performance.

[13] Bidet-Caulet A, Mikyska C, Knight RT (2010). Load effects in auditory selective attention: Evidence for distinct facilitation and inhibition mechanisms. NeuroImage.

[14] Michie PT, Solowij N, Crawford JM, Glue LC (1993). The effects of between‐source discriminability on attended and unattended auditory ERPs. Psychophysiology.

[15] Tong Y, Melara RD, Rao A (2009). P2 enhancement from auditory discrimination training is associated with improved reaction times. Brain research.

[16] Allison T, Puce A, McCarthy G (2002). Category-sensitive excitatory and inhibitory processes in human extrastriate cortex. Journal of neurophysiology.

[17] Näätänen, R. *Attention and brain function*. (Psychology Press, 1992).

[18] Hillyard SA, Hink RF, Schwent VL, Picton TW (1973). Electrical signs of selective attention in the human brain. Science.

[19] Näätänen R, Gaillard AW, Mäntysalo S (1978). Early selective-attention effect on evoked potential reinterpreted. Acta psychologica.

[20] Rif J, Hari R, Hämäläinen MS, Sams M (1991). Auditory attention affects two different areas in the human supratemporal cortex. Electroencephalography and clinical neurophysiology.

[21] Choi I, Wang L, Bharadwaj H, Shinn-Cunningham B (2014). Individual differences in attentional modulation of cortical responses correlate with selective attention performance. Hearing research.

[22] Coenen A (2012). Modelling of auditory evoked potentials of human sleep-wake states. International journal of psychophysiology: official journal of the International Organization of Psychophysiology.

[23] Ogilvie RD, Simons IA, Kuderian RH, MacDonald T, Rustenburg J (1991). Behavioral, Event‐Related Potential, and EEG/FFT Changes at Sleep Onset. Psychophysiology.

[24] Campbell KB, Colrain IM (2002). Event-related potential measures of the inhibition of information processing: II. The sleep onset period. International Journal of Psychophysiology.

[25] Huang J (2017). Evoked potentials and behavioral performance during different states of brain arousal. BMC neuroscience.

[26] de Lugt DR, Loewy DH, Campbell KB (1996). The effect of sleep onset on event related potentials with rapid rates of stimulus presentation. Electroencephalography and clinical neurophysiology.

[27] Coenen A (2003). Auditory evoked potentials of sleep-wake states in humans: a qualitative psychophysiological interpretation. Sleep-Wake Research in the Netherlands.

[28] Steriade M, McCormick DA, Sejnowski TJ (1993). Thalamocortical oscillations in the sleeping and aroused brain. Science.

[29] Edeline JM, Manunta Y, Hennevin E (2000). Auditory thalamus neurons during sleep: changes in frequency selectivity, threshold, and receptive field size. Journal of neurophysiology.

[30] Coenen AM, Vendrik AJ (1972). Determination of the transfer ratio of cat’s geniculate neurons through quasi-intracellular recordings and the relation with the level of alertness. Experimental brain research.

[31] Isaacson JS, Scanziani M (2011). How inhibition shapes cortical activity. Neuron.

[32] Chellappa, S. L. *et al*. Circadian dynamics in measures of cortical excitation and inhibition balance. *Scientific Reports* **6** (2016). 10.1038/srep33661 PMC5030482 27651114

[33] Colonnese MT (2014). Rapid developmental emergence of stable depolarization during wakefulness by inhibitory balancing of cortical network excitability. Journal of Neuroscience.

[34] Marín O (2012). Interneuron dysfunction in psychiatric disorders. Nature Reviews Neuroscience.

[35] Gogolla N (2009). Common circuit defect of excitatory-inhibitory balance in mouse models of autism. Journal of neurodevelopmental disorders.

[36] Yang C, Lo H (2007). ERP evidence of enhanced excitatory and reduced inhibitory processes of auditory stimuli during sleep in patients with primary insomnia. Sleep.

[37] Yao S (2010). Inhibition dysfunction in depression: Event-related potentials during negative affective priming. Psychiatry Research: Neuroimaging.

[38] Bajbouj M (2006). Evidence for impaired cortical inhibition in patients with unipolar major depression. Biological psychiatry.

[39] Schubert J (2014). Dysfunctional cortical inhibition in adult ADHD: Neural correlates in auditory event-related potentials. Journal of neuroscience methods.

[40] Turcotte I, Bastien CH (2009). Is quality of sleep related to the N1 and P2 ERPs in chronic psychophysiological insomnia sufferers?. International Journal of Psychophysiology.

[41] Cortoos A, De Valck E, Pattyn N, Mairesse O, Cluydts R (2014). Excitatory versus inhibitory impairments in insomnia patients: An ERP study. International Journal of Psychophysiology.

[42] Bonnet MH (2010). Hyperarousal and insomnia. Sleep medicine reviews.

[43] Riemann D (2010). The hyperarousal model of insomnia: a review of the concept and its evidence. Sleep medicine reviews.

[44] Hegerl U, Wilk K, Olbrich S, Schoenknecht P, Sander C (2012). Hyperstable regulation of vigilance in patients with major depressive disorder. The world journal of biological psychiatry: the official journal of the World Federation of Societies of Biological Psychiatry.

[45] Schwabedal JT, Riedl M, Penzel T, Wessel N (2016). Alpha‐wave frequency characteristics in health and insomnia during sleep. Journal of sleep research.

[46] Ulke, C. *et al*. Sleep disturbances and upregulation of brain arousal during daytime in depressed versus non-depressed elderly subjects. *The world journal of biological psychiatry: the official journal of the World Federation of Societies of Biological Psychiatry*, 1–21, doi:10.1080/15622975.2016.1224924 (2016). 10.1080/15622975.2016.1224924 27557150

[47] Olbrich S (2016). CNS-and ANS-arousal predict response to antidepressant medication: Findings from the randomized iSPOT-D study. Journal of psychiatric research.

[48] Tenke CE (2011). Current source density measures of electroencephalographic alpha predict antidepressant treatment response. Biological psychiatry.

[49] Bruder, G. E., Tenke, C. E. & Kayser, J. Electrophysiological predictors of clinical response to antidepressants. *The Clinical Handbook for the Management of Mood Disorders*, 380–393 (2013).

[50] Benarroch EE (2009). The locus ceruleus norepinephrine system Functional organization and potential clinical significance. Neurology.

[51] Carter ME, de Lecea L, Adamantidis A (2013). Functional wiring of hypocretin and LC-NE neurons: implications for arousal. Frontiers in behavioral neuroscience.

[52] Aston-Jones, G., Gonzalez, M. & Doran, S. Role of the locus coeruleus-norepinephrine system in arousal and circadian regulation of the sleep–wake cycle. *Brain norepinephrine: Neurobiology and therapeutics*, 157–195 (2007).

[53] Goddard AW (2010). Current perspectives of the roles of the central norepinephrine system in anxiety and depression. Depression and anxiety.

[54] Hegerl U, Hensch T (2014). The vigilance regulation model of affective disorders and ADHD. Neuroscience and biobehavioral reviews.

[55] Nieuwenhuis S, De Geus EJ, Aston‐Jones G (2011). The anatomical and functional relationship between the P3 and autonomic components of the orienting response. Psychophysiology.

[56] Walz JM (2013). Simultaneous EEG-fMRI reveals temporal evolution of coupling between supramodal cortical attention networks and the brainstem. The Journal of neuroscience: the official journal of the Society for Neuroscience.

[57] Breeden A, Siegle G, Norr M, Gordon E, Vaidya C (2016). Coupling between spontaneous pupillary fluctuations and brain activity relates to inattentiveness. European Journal of Neuroscience.

[58] Bonnet M, Arand D (1997). Heart rate variability: sleep stage, time of night, and arousal influences. Electroencephalography and clinical neurophysiology.

[59] Brandenberger G, Ehrhart J, Piquard F, Simon C (2001). Inverse coupling between ultradian oscillations in delta wave activity and heart rate variability during sleep. Clinical neurophysiology.

[60] Yuan H, Zotev V, Phillips R, Bodurka J (2013). Correlated slow fluctuations in respiration, EEG, and BOLD fMRI. NeuroImage.

[61] Long X (2015). Time delay between cardiac and brain activity during sleep transitions. Applied Physics Letters.

[62] Bastuji H, García-Larrea L (1999). Evoked potentials as a tool for the investigation of human sleep. Sleep medicine reviews.

[63] Boutros NN, Korzyukov O, Jansen B, Feingold A, Bell M (2004). Sensory gating deficits during the mid-latency phase of information processing in medicated schizophrenia patients. Psychiatry research.

[64] Lőrincz ML, Geall F, Bao Y, Crunelli V, Hughes SW (2009). ATP-dependent infra-slow (<0.1 Hz) oscillations in thalamic networks. PloS one.

[65] Steriade M (2006). Grouping of brain rhythms in corticothalamic systems. Neuroscience.

[66] Schwabedal JT, Knapper DE, Shilnikov AL (2016). Qualitative and quantitative stability analysis of penta-rhythmic circuits. Nonlinearity.

[67] Lu J, Sherman D, Devor M, Saper CB (2006). A putative flip–flop switch for control of REM sleep. Nature.

[68] Rye DB (2012). Modulation of vigilance in the primary hypersomnias by endogenous enhancement of GABAA receptors. Science translational medicine.

[69] Stefanovska A (2007). Coupled oscillatros: complex but not complicated cardiovascular and brain interactions. IEEE Engineering in Medicine and Biology Magazine.

[70] Kvernmo HD, Stefanovska A, Kirkeboen KA, Kvernebo K (1999). Oscillations in the human cutaneous blood perfusion signal modified by endothelium-dependent and endothelium-independent vasodilators. Microvasc Res.

[71] Soderstrom T, Stefanovska A, Veber M, Svensson H (2003). Involvement of sympathetic nerve activity in skin blood flow oscillations in humans. American journal of physiology. Heart and circulatory physiology.

[72] Kvandal P (2006). Low-frequency oscillations of the laser Doppler perfusion signal in human skin. Microvascular research.

[73] Olbrich S (2009). EEG-vigilance and BOLD effect during simultaneous EEG/fMRI measurement. NeuroImage.

[74] Moore CI, Cao R (2008). The hemo-neural hypothesis: on the role of blood flow in information processing. Journal of neurophysiology.

[75] Saper CB, Chou TC, Scammell TE (2001). The sleep switch: hypothalamic control of sleep and wakefulness. Trends in neurosciences.

[76] Åkerstedt T, Gillberg M (1990). Subjective and objective sleepiness in the active individual. International Journal of Neuroscience.

[77] Squires NK, Squires KC, Hillyard SA (1975). Two varieties of long-latency positive waves evoked by unpredictable auditory stimuli in man. Electroencephalography and clinical neurophysiology.

